# MiR133b‐mediated inhibition of EGFR‐PTK pathway promotes rAAV2 transduction by facilitating intracellular trafficking and augmenting second‐strand synthesis

**DOI:** 10.1111/jcmm.17858

**Published:** 2023-07-19

**Authors:** Xiaoping Huang, Xiao Wang, Ling Li, Qizhao Wang, Wentao Xu, Wenlin Wu, Xiaolan Xie, Yong Diao

**Affiliations:** ^1^ College of Chemical Engineering and Materials Sciences Quanzhou Normal University Quanzhou China; ^2^ School of Medicine Huaqiao University Quanzhou China

**Keywords:** EGFR‐protein tyrosine kinase, miRNA133b, rAAV2, second‐strand DNA synthesis, ubiquitination

## Abstract

Recombinant adeno‐associated virus (rAAV) is an extremely attractive vector in the in vivo delivery of gene therapy as it is safe and its genome is simple. However, challenges including low permissiveness to specific cells and restricted tissue specificity have hindered its clinical application. Based on the previous studies, epidermal growth factor receptor‐protein tyrosine kinase (EGFR‐PTK) negatively regulated rAAV transduction, and EGFR‐positive cells were hardly permissive to rAAV transduction. We constructed a novel rAAV‐miRNA133b vector, which co‐expressed miRNA133b and transgene, and investigated its in vivo and in vitro transduction efficiency. Confocal microscopy, live‐cell imaging, pharmacological reagents and labelled virion tracking were used to analyse the effect of miRNA133b on rAAV2 transduction and the underlying mechanisms. The results demonstrated that miRNA133b could promote rAAV2 transduction and the effects were limited to EGFR‐positive cells. The increased transduction was found to be a direct result of decreased rAAV particles degradation in the cytoplasm and enhanced second‐strand synthesis. ss‐rAAV2‐miRNA133b vector specifically increased rAAV2 transduction in EGFR‐positive cells or tissues, while ss‐rAAV2‐Fluc‐miRNA133b exerted an antitumor effect. rAAV‐miRNA133b vector might emerge as a promising platform for delivering various transgene to treat EGFR‐positive cell‐related diseases, such as non‐small‐cell lung cancer.

## 1 INTRODUCTION

Adeno‐associated virus (AAV) is the single‐strand, nonenveloped DNA virus belonging to Parvoviridae family. It has been categorized into the Dependovirus because it demands one helper virus for replication, like herpes simplex virus (HSV) or adenovirus.^1^, ^2^, ^3^ AAV genome persists episomally without the helper virus.^4^, ^5^ Recombinant AAVs (rAAVs) represent the key gene therapy vectors, which have been clinically used in gene therapy. Alipogene tiparvovec (Glybera), the first gene therapy drug production based on rAAV1, was approved by the European Medicines Agency in 2012, and aimed against lipoprotein lipase deficiency. Five years later, the United States approved voretigene neparvovec‐rzyl (Luxturna) for Leber's congenital amaurosis.^6^, ^7^, ^8^ Although rAAV vectors can be used for in vivo gene therapy, insufficient transduction efficiency in certain cells or tissues is a limitation of their clinic applications.^9^, ^10^, ^11^ For example, intravenous administration of rAAV vectors is commonly used for liver‐based hereditary diseases, because of their liver tropism; however, the rAAV mediated transgene expression was very low within several subtypes of hepatocytes due to their high expression of epidermal growth factor receptor (EGFR).^12^

The EGFR‐protein tyrosine kinase (EGFR‐PTK) is reported to exert an important role in negatively regulating rAAV transduction.^13^, ^14^, ^15^ EGFR‐PTK can phosphorylate FK506‐binding protein (FKBP52), while the phosphorylated form of FKBP52 interacts with the D‐sequence in the inverted terminal repeat (ITR) of rAAV genome and inhibits its second‐strand DNA synthesis.^15^, ^16^ The single‐stranded DNAs packaged in rAAV vectors must be converted to double‐stranded DNA (dsDNA) to be transcriptionally active. Therefore, EGFR‐PTK negatively regulates rAAV transduction by inhibiting its viral second‐strand synthesis.^17^ The efficiency of rAAV intracellular trafficking is closely correlated with the ubiquitin (Ub)‐proteasome pathway. rAAV capsids can be phosphorylated by EGFR‐PTK after they escape from late endosome and expose surface tyrosine residues in the cytoplasm. Subsequently, the phosphorylated rAAV vectors are ubiquitinated and degraded by the cytoplasm proteasome. Therefore, it is possible to promote the transduction efficiency of rAAV in EGFP‐positive cells by regulating the activity of EGFR‐PTK.

In the field of AAV capsid engineering, great efforts have been made to improve rAAV transduction, including directing evolution of the capsid, site‐directing mutagenesis and peptide insertion of capsid and random chimeras of capsid.^18^, ^19^, ^20^, ^21^ Although rAAV capsid engineering field has been under flourishing development, and related techniques have been increasing quickly, only a few efforts are made to improve recombinant genome that encodes the DNA of interest.^22^ One notable example is the construction of self‐complementary (sc) rAAV vector, in which dsDNA genome is packaged to express transgene faster and more potently than the conventional ddDNA counterpart. Incorporating 4 miR‐142‐3p binding sites (miRNA‐BS) in 3'UTR in rAAV genome led to specific downregulation of transgen in splenocytes rather than hepatocytes.^23^ Many microRNAs have been used for regulating AAV transgene expression.^22^ miRNA133b regulates the EGFR‐PTK activity in several non‐small‐cell lung cancer (NSCLC) cell lines. Moreover, miRNA133b also affects NSCLC cell proliferation, migration, invasion and apoptosis.^24^ Based on the above analysis, we hypothesize that miRNA133b may promote the transduction of rAAV in EGFR‐positive cells by regulating EGFR‐PTK signalling.

This study analysed miRNA133b's function in rAAV2 transduction and the underlying mechanisms by several technologies, like live‐cell imaging, confocal microscopy, labelled virion tracking and pharmacological reagents. Based on live‐cell imaging, rAAV2 transduction increased when there was no miRNA133b, and the effects were limited to EGFR expressing cells. These results were further supported by the measurement of real‐time PCR and labelled virion tracking, indicating that both steps of intracellular trafficking and second‐strand synthesis of rAAV2 in EGFR‐positive cell were significantly increased after miRNA133b pretreatment. For the first time, we have demonstrated that the incorporation of miRNA133b in the rAAV2 genome specifically increases transgene expression in EGFR‐positive cells, and can be used as an alternative technology to improve the transduction features of rAAV vectors.

## 2 MATERIALS AND METHODS

### 2.1 Cell culture and chemicals

In this study, 293T (ATCC, CRL‐3216) cells were inoculated in DMEM (Gibco), while HeLa (ATCC, CCL‐2), A549 (ATCC, CCL‐185) and NCI‐H446 (ATCC, HTB‐171) cells were inoculated in RPMI‐1640 (Gibco), and CHO (National Collection of Authenticated Cell Cultures, SCSP‐507) cells were inoculated in F‐12 Kaighn's (Thermo Fisher Scientific). The above cell lines were kept at 37°C with 5% CO_2_. The media contained 1% penicillin–streptomycin (PS) and 10% foetal bovine serum (FBS). MG132 (Sigma Aldrich), a proteasome inhibitor, was also added into the dimethyl sulphoxide (10 mM, DMSO). Gefitinib (Beyotime) was prepared to 1 mM in DMSO. The miRNA133b mimic and miRNA scramble (Gene Pharma) were performed at 15 nM with Lipofectamine 2000 (Thermo Fisher Scientific).

### 2.2 Recombinant AAV2 production

The rAAV2 vector was produced in 293T cells. Briefly, adenovirus helper, capsid and transgenic plasmids transfected 293T cells to produce rAAV2. After 72 h, transfected cells were subjected to collection, lysis and treatment with DNase. After vector purification at the gradient concentrations of caesium chloride and dialysis for removing caesium chloride, quantitative PCR (qPCR) was performed to determine vector titres according to the previous description.^25^

### 2.3 Transfection of recombinant AAV2 vector

HeLa, NCI‐H446, A549 and CHO cells (1 × 10^4^/well) were inoculated into the 96‐well plate for 12 h incubation at 37°C. After exposure to miRNA133b, MG132 and gefitinib, cells were subjected to different multiplicities of rAAV2 infected cells for 72 h at 37°C. The transduction efficiency and GFP expression were monitored by the IncuCyte cell imaging system (Essen BioScience). This system was also used to analyse fluorescence intensity in three fields of each well. The Gaussia Luciferase Assay Kit (Beyotime) was employed to determine the *Gaussia luciferase* expression in cultivating supernatants.

### 2.4 Capsid labelling and confocal microscopy

Confocal imaging of tetramethylrhodamine (TAMRA)‐labelled rAAV2 was performed in line with the previous description.^26^ In brief, mono‐NHS‐TAMRA molecules/vector genome (Sangon Biotech) were added to incubate pure rAAV2 virions at room temperature for 45 min. Then, the SpinOUT™ GT‐600 column (G‐Biosciences) removed the unconjugated dye. After labelling, qPCR determined vector titre. For confocal imaging experiments, miRNA133b transfected HeLa cells for 24 h, and the TAMRA‐conjugated rAAV2 were mixed with those transfected cells (2000 VG/cell). The rAAV2 distribution was analysed at 6, 12 and 24 h, respectively. At harvest, after rinsing three times by PBS, cells were fixed with 4% paraformaldehyde (PFA) for 15 min at room temperature, followed by washing three times using PBS and double‐distilled H_2_O. Subsequently, cells were dyed by 4,6‐diamidino‐2‐phenylindole (DAPI) (Sigma Aldrich). The Zeiss LSM 710 spectral confocal laser‐scanning microscope (Zeiss) was used to analyse rAAV2 virion location at 10^4^ particles/cell.

### 2.5 Cellular cytoplasmic and nuclear fraction separation

HeLa cells were applied to separate their cytoplasmic and nuclear fractions according to the previous description.^27^ Briefly, rAAV2‐GFP infected cells at 5000 particles/cell were treated with 0.01% trypsin treatment, and then washed five times by PBS to remove unabsorbed viral particles. Thereafter, hypotonic buffer (200 μL, comprising of 10 mM KCl, 10 mM HEPES, pH 7.9, 1.5 mM MgCl_2_, 0.5 mM phenylmethanesulphonyl fluoride, 0.5 mM dithiothreitol) was added to resuspend cell pellets, followed by 5 min on ice. Next, 10% NP‐40 (10 μL) was added to the tube for about 3 min and then observed under the light microscope (Nikon). After gentle mixing, samples were centrifuged for 5 min at 500 rpm at 4°C. Next, the cytoplasmic fraction (supernatant) was added slowly before preservation on ice, while the nuclear fraction (pellet) was rinsed by hypotonic buffer (1 mL) before being preserved on ice. Histone H3 (cytoplasmic fraction) and acid phosphatase activity (nuclear fraction) determined purity of each fraction as previously described.^28^, ^29^ The nuclear and cytoplasmic purity was >95%.

### 2.6 Whole‐cell lysate (WCL) preparation and co‐immunoprecipitation (CO‐IP) assay

WCL was made according to previous description after the following modifications below^30^, ^31^, ^32^: MG132 and/or miRNA133b were used to treat HeLa cells (2 × 10^6^). To analyse cell proteins, cell lysis buffer (10% glycerol, 1% Triton X‐100, 150 mM NaCl, 50 mM HEPES, pH 7.5, 1 mM EDTA 1.5 mM MgCl_2_) containing 10 mM NaF, 1 mM dithiothreitol, 0.5 mM phenylmethanesulphonylfluoride, 2 mM Na3VO4, 10 μg/mL pepstatin, 10 μg/mL leupeptin and 10 μg/mL aprotinin was added to lyse cells on ice. In CO‐IP assay, HeLa cells were exposed to MG132 or miRNA133b, or both, followed by mock‐infection or infection by rAAV2‐GFP vectors for 6 h at 37°C at 10^4^ particles/cell. Later, 0.01% trypsin was supplemented to treat cells, and washed by PBS for 4 h post‐infection in order to remove unabsorbed virus particles and resuspension in the hypotonic buffer (2 mL). WCL was made using cell homogenisation method in the tight‐fitting dual tissue grinder (Beyotime) until approximately 95% cells were lysed. This was monitored with the trypan blue dye (Sigma Aldrich) exclusion assay. Incubation involved normal mouse IgG (0.25 μg) and protein G‐agarose beads (20 μL) for 60 min at 4°C in the orbital shaker cleared WCL of non‐specific binding. After normal mouse IgG clearing, we added capsid antibody (2 μg) to resist intact mouse IgG1 AAV2 (A20), followed by 60 min incubation at 4°C. The particles were precipitated using protein G‐agarose beads for 12 h at 4°C in the shaker. After the collection, pellets were centrifuged for 5 min at 2500 rpm and 4°C, followed by four washes with PBS. Supernatants from the last washing were discarded, followed by resuspension of the pellets with 2 μL sodium dodecyl sulphate (SDS) solution. Based on the above description, pellet solution (20 μL) was used in western blotting (WB) assay.

### 2.7 RNA preparation, miRNA133b qPCR analysis

The HeLa cells were transduced with ss‐rAAV2‐GFP or ss‐rAAV2‐GFP‐miRNA133b in 6‐well plates for 48 h. We utilized Trizol for total RNA extraction, which was later prepared into cDNA by Primer Script RT reagent Kit (TaKaRa Bio) via reverse transcription. All the experimental procedures were performed at low temperature. The miRNA expression was examined using AceQ qPCR SYBR Green Master Mix (SYBR Green I) kit (Vazyme Biotechnology) with an ABI Step One Fast Real‐Time PCR System by applying gene‐specific primers (Table S1) in line with specific protocols. Relative content of miRNA was normalized to endogenous U6 small nuclear RNA.

### 2.8 DNA purification and qPCR on viral genome copy number

rAAV2 was transfected into HeLa cells at 5000 vgs/cell for 12 h, followed by isolation of cytoplasmic and nuclear fractions. In line with specific protocols, total DNA of cytoplasmic and nuclear fractions were collected with Qiagen DNeasy blood and tissue kit. Human GAPDH gene was the nuclear reference genes. Cell internal reference genes and viral genomes were analysed based on the previous description.^33^ We used all primers (Table S1) for quantifying GFP, EGFR and GAPDH. All the reactions were run with AceQ qPCR SYBR Green Master Mix (SYBR Green I) kit (Vazyme Biotechnology) on ABI Step One Fast Real‐Time PCR System. PCR conditions were shown as following: 10 min at 95°C; 10 s at 95°C, 10 s at 60°C and 10 s at 72°C for 45 cycles. For absolute quantification, second‐derivative maximum comparisons were made against the plasmid DNA standard curves.

### 2.9 Bioinformatics analysis and luciferase reporter assay

PICTAR (http://pictar.mdc‐berlin.de/) and TARGETSCAN (http://www.targetscan.org/) websites were searched to identify the possible targets of miR‐133b on EGFR (accession no. NM_005228). 3′‐UTR (approximately 100‐bp) in human EGFR mRNA encompassing miRNA133b seed sequence‐complementary nucleotide sequence was inserted in psiCHECK miRNA Expression Reporter Vector System. Then, cDNA was prepared from 1 μg total RNA through the above‐mentioned reverse transcription, followed by amplification of 3′‐UTR using primers below: 5′‐cgctcgagccacgg aggata gtatgag‐3′ (Forward); 5′‐cagcggccgcagctaatgcgggcatgg c t‐3′ (Reverse). *Not* I and *Xho* I (NEB; New England Biolabs) were used for digestion of purified DNA. The psiCHECK‐EGFR‐3’UTR‐Luc plasmid (reporter plasmid) was constructed to verify 3′‐UTR DNA sequence. To perform luciferase assay, HeLa cells (2 × 10^5^/well) were added into the 96‐well plate for overnight culture. On Day 2, 0.1 μg psiCHECK‐EGFR‐3′UTR plasmid was co‐transfected with miRNA133b mimic or scrambled miRNA, using Lipofectamine 2000 (Invitrogen). After 48 h, cells were subject to lysis and analysis of luciferase activities (firefly‐to‐Renilla luciferase activity ratio) using the Dual‐Luciferase Assay reagent (Promega). In addition, all the procedures were conducted three times in three separate assays.

### 2.10 ss‐rAAV2 second‐strand DNA synthesis

rAAV2 second‐strand DNA was synthesized with a Click‐iTEdU Alexa Fluor 594 Kit (Ribobio) in line with the specific protocols. We inoculated approximately 6 × 10^5^ HeLa cells into each well, followed by 12 h incubation at 37°C, pretreatment using miRNA133b for 24 h, transfection with ss‐rAAV2 and 36‐h incubation using 2 μM EdU at 37°C. Then, 4% PFA was added to fix cells for 15 min, followed by permeabilisation for 20 min using 0.5% Triton X‐100 at room temperature. After washing three times with PBS, Apollo reaction cocktail was added to incubate cells for 30 min. Viral genomic Hirt DNA was extracted from the cells, and the viral genomic Hirt DNA fluorescence was determined by fluorescence spectroscopy (HITACHI, F‐7000).

### 2.11 Hirt DNA purification

Cells were washed once with pre‐chilled DPBS in 60 mm plates, followed by lysis with Hirt lysis buffer (500 μL, comprising 0.6% SDS, 10 mM EDTA (pH 7.5), 10 mM Tris). Later, cells were incubated for 10 min at room temperature, followed by addition of 5 M NaCl (120 μL) and 2 min gentle rocking. A cell scraper was used to scrape cell lysate in the plate, which was later added into the microcentrifuge tube to incubate overnight at 4°C. Afterwards, cells were centrifuged for 40 min at 17,000 g at 4°C, followed by pellet removal and addition of 20 mg/mL proteinase K (1.25 μL) into supernatant to incubate for 60 min at 37°C. After overnight incubation at 4°C, cells were centrifuged for 60 min at 17,000 g and 4°C. Supernatants were collected into a new tube. Saturated phenol was added to extract low molecular weight DNA three times, while chloroform/isoamyl alcohol was added to extract once. This was followed by 3 h precipitation of viral DNA at −80°C after ethanol (twofold volume) was added together with 60 min centrifugation at 17,000 g and 4°C. After being washed by 70% ethanol, pellets were centrifuged for 5 min centrifugation, followed by DNA drying and pellet suspension in the double‐distilled H_2_O (100 μL).

### 2.12 In vivo tumour xenografts

Each animal experiment was approved by Institutional Animal Care and Use Committee (No. AHQU018016) and performed following animal care guidelines released by Animal Care Services of Huaqiao University (Quanzhou, China). Pain and animal sufferings were minimized. We obtained the 6‐ to 8‐week‐old BALB/C mice in Shanghai Laboratory Animal Central (SLAC). Later, BALB/C mice were inoculated with 1 × 10^6^/50 μL NCI‐H446 cells via subcutaneous inoculation. After 7 days when tumour grew to a volume of around 50 mm^3^ (V = a × b^2^/2, in which a and b stand for length and width, respectively), we randomized mice as treatment groups (*n* = 3 each). Later, PBS (100 μL) was added to dilute ss‐rAAV‐Fluc and ss‐rAAV‐Fluc‐miRNA133b, followed by intravenous injection at 10^11^ vg/mouse in tail vein. Live imaging of ss‐rAAV2‐Fluc‐miRNA133b and ss‐rAAV2‐Fluc vector luciferase activity was previously depicted. D‐luciferin (Thermo Fisher Scientific) was intraperitoneally injected (150 mg/kg) into each mouse. An IVIS‐Lumina imaging system (FluoView 100, Guangzhou Biolight Biotechnology Co., Ltd) was utilized to determine bioluminescence activity. Living Image software was employed to quantify the signal intensity of bioluminescence, which was shown in units of photons/s/cm^2^/steradian. Mice were killed following tumour growth for 28 days. Transgene expression in tumours and organs was analysed.

### 2.13 Plasmids

To generate the pLV‐TC‐PTP construct, pLV was linearized using enzymes *Bam*H I and *Mlu* I. TC‐PTP DNA fragment was synthesized. TC‐PTP DNA was cloned into pLV and then transformed into TOP10 Escherichia coli. To generate the pAAV‐GFP‐U6‐miRNA133b construct, the U6 promoter was first cloned into pAAV‐GFP. Then, pAAV‐GFP‐U6 was linearized using the enzymes *Hind* III and *Not* I. PremiRNA133b was synthesized and later amplified by PCR. The premiRNA133b DNA was cloned into pAAV‐GFP‐U6. Table S2 shows PCR primers used for plasmid construction.

### 2.14 Western blot assay

WB assay was performed according to the previous description [30]. To investigate cellular proteins, about 5 μg of WCL was isolated using 10% SDS‐PAGE, followed by transfer onto PVDF membranes (Millipore). The resuspended pellet solution was heated at 100°C for 2–3 min before the samples (20 μL) were resolved using SDS‐PAGE. Afterwards, 5% defatted milk contained in Tris‐buffered saline (150 mM NaCl, 20 mM Tris–HCl, pH 7.5) was added to block membranes for 12 h at 4°C, followed by overnight incubation at 4°C using indicated primary antibodies shown below: rabbit anti‐FKBP52 (#11826, CST), rabbit anti‐Phospho‐FKBP52 (#AF7065, Affinity), rabbit anti‐TC‐PTP (#58935, CST), rabbit‐EGFR (#4267, CST), mouse‐Ubiquitin (#3936, CST), mouse anti‐AAV2 A20 antibody (03‐61055, ARP) and rabbit anti‐GAPDH. Later, membranes were rinsed before being incubated using horseradish peroxidase (HRP)‐conjugated anti‐rabbit (1:1000; R & D Systems) or horseradish peroxidase‐labelled anti‐mouse (1:1000; R & D Systems) under room temperature for 60 min. Then, an ECL–plus chemiluminescence substrate (Amersham Pharmacia Biotech) was used for detecting protein bands. Image J was applied to quantify signal intensity.

### 2.15 Statistical analysis

The results were represented by mean ± SD. Student's *t*‐test was performed for data statistical analysis. One‐way anova was applied to analyse cell growth, apoptosis, qRT‐PCR and WB assays. *p* < 0.05 (two‐sided) represented statistical significance. GRAPHPAD v5.0 (GraphPad) and SPSSv13.0 (SPSS) were used in the statistical analysis.

## 3 RESULTS

### 3.1 miRNA133b increases rAAV2 transduction in EGFR‐positive cells

It was shown that rAAV2 transduction efficiency associated with EGFR expression inversely.^15^ Therefore, we supposed that inhibiting EGFR expression with miRNA will be beneficial for rAAV2 transduction. miRNA133b, which contains seven‐nucleotide seed sequence matching to EGFR mRNA 3′ untranslated region (3’UTR) at positions 50–56 (Figure S1A), was selected for downregulating EGFR. To confirm whether miRNA133b is functional in inhibiting EGFR expression in HeLa cells, EGFR 3′UTR Luciferase Reporter Assay was performed. Transfection of miRNA133b inhibited the luciferase reporter gene levels, and it was under the regulation of EGFR 3′UTR in the reporter plasmid, while scrambled miRNA did not (Figure S1B). In addition, WB results also confirmed that EGFR levels in HeLa cells decreased with miRNA133b (Figure S1C).

**FIGURE 1:**
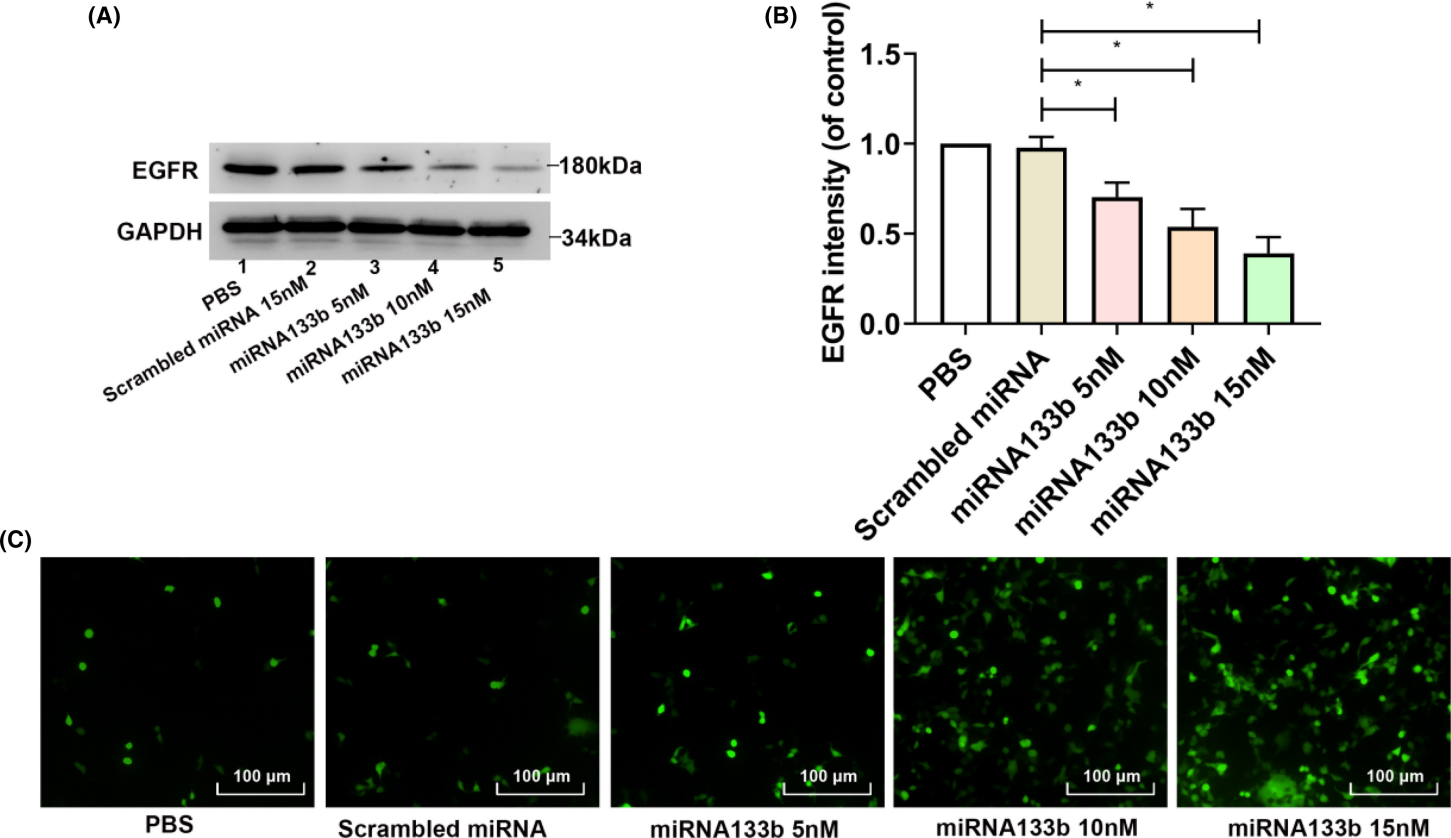
Role of epidermal growth factor receptor (EGFR) in rAAV2 transfection. (A) WB assay on EGFR levels in HeLa cells after transfection using scrambled miRNA and miRNA133b, (B) EGFR expression was analysed through densitometry compared with GAPDH. Results are shown by mean ± SD (*N* = 3). *p < 0.05. (C) The representative images of HeLa cells transfected with scrambled miRNA or miRNA133b and then transduced with ss‐rAAV2 for 48 h.

Then, the potential functionality of miRNA133b on rAAV2 transduction was evaluated. HeLa cells were pretreated with miRNA133b at a range of doses, and then transduced with ss‐rAAV2‐GFP (1500 vg/cell) vectors, with scrambled miRNA as the control. HeLa cells are EGFR‐positive cell line, and have been commonly used for rAAV transduction. A dose‐dependent reduction of EGFR expression in HeLa cells was confirmed (Figure 1A,B), in relative to untreated control. GFP levels were positively correlated with miRNA133b concentrations (Figure 1C). The dose of 15 nM miRNA133b was used in the following experiments. The results suggest that miRNA133b treatment has the potential to increase rAAV2 transduction.

**FIGURE 2:**
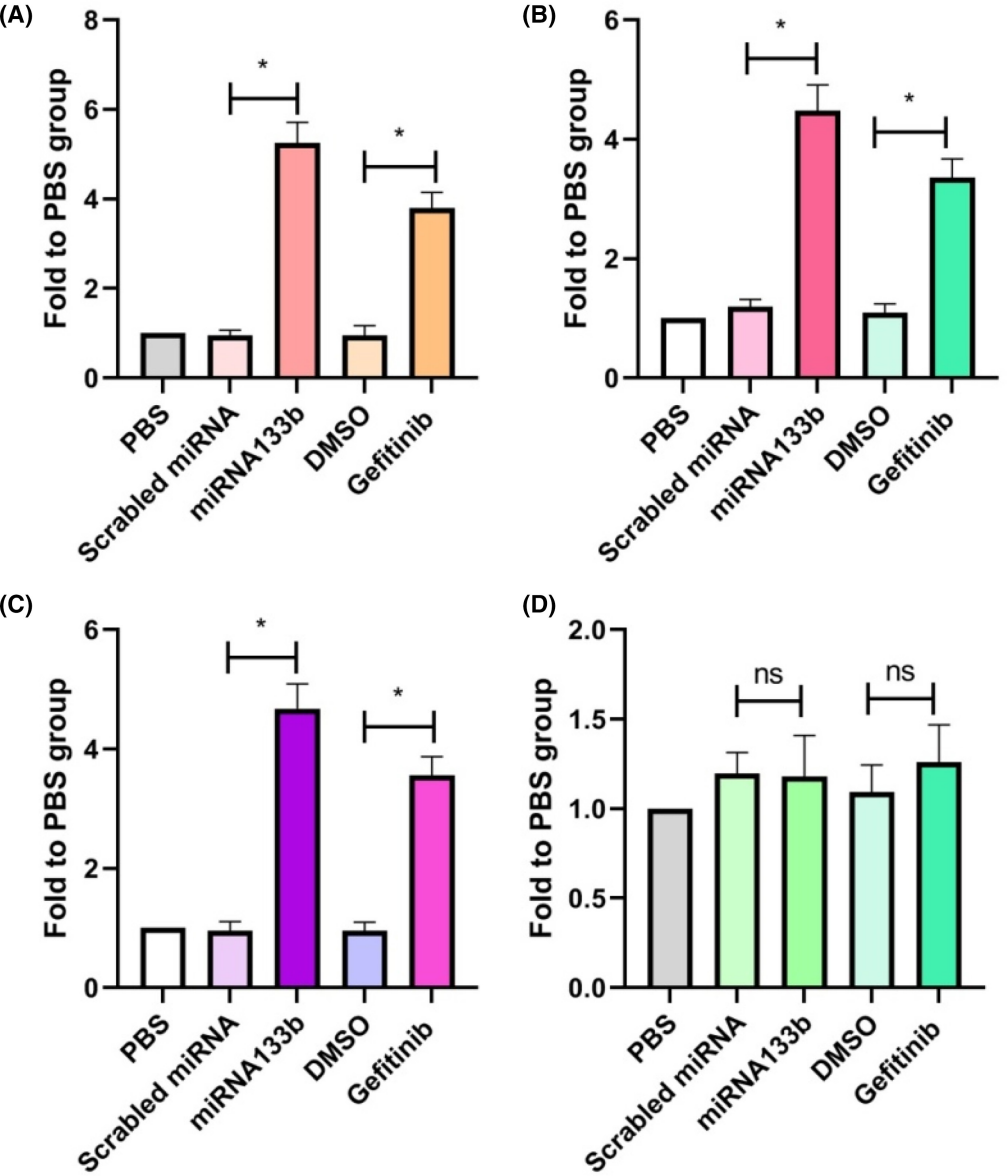
ss‐rAAV2‐luciferase transduction after miRNA133b or gefitinib exposure in multiple non‐human and human cells. Hep3B (A), NCI‐H446 (B), A549 (C) and CHO‐K1 (D) cells treated with 15 nM miRNA133b or 5 μM gefitinib were transduced with ss‐rAAV2‐Luc. Luciferase expression was measured by a luminometer at 48 h post‐transduction. All values are shown as means ± SD (*N* = 3). *p < 0.05, ^ns^ *p* > 0.05.

To analyse whether the role of miRNA133b in rAAV2 transfection was restricted into EGFR‐positive cells, several different cell lines were tested. These cells were subjected to overnight treatment using miRNA133b (15 nM), transfection using ss‐rAAV2‐Luc, as well as the analysis of luciferase expression at 48 h post‐transfection. In Hep3B, NCI‐H446 and A549, which are all EGFR‐positive cells, miRNA133b treatment increased the luciferase activities after ss‐rAAV2‐Luc transduction 5.2, 4.8 and 4.4 times in relative to PBS group (Figure 2A–D). By contrast, the luciferase activity in the CHO‐K1 cell line was not significantly changed after miRNA133b treatment, in relative to either PBS or scrambled miRNA group. Gefitinib did not increase the transgene expression either, compared with DMSO treatment (Figure 2D). The results suggest that miRNA133b increases rAAV2 transduction only in EGFR expression positive cells, and that miRNA133b has a critical effect on rAAV2 transfection in EGFR expression positive cells.

### 3.2 miRNA133b increases both ss‐rAAV2 and sc‐rAAV2 transduction

**FIGURE 3:**
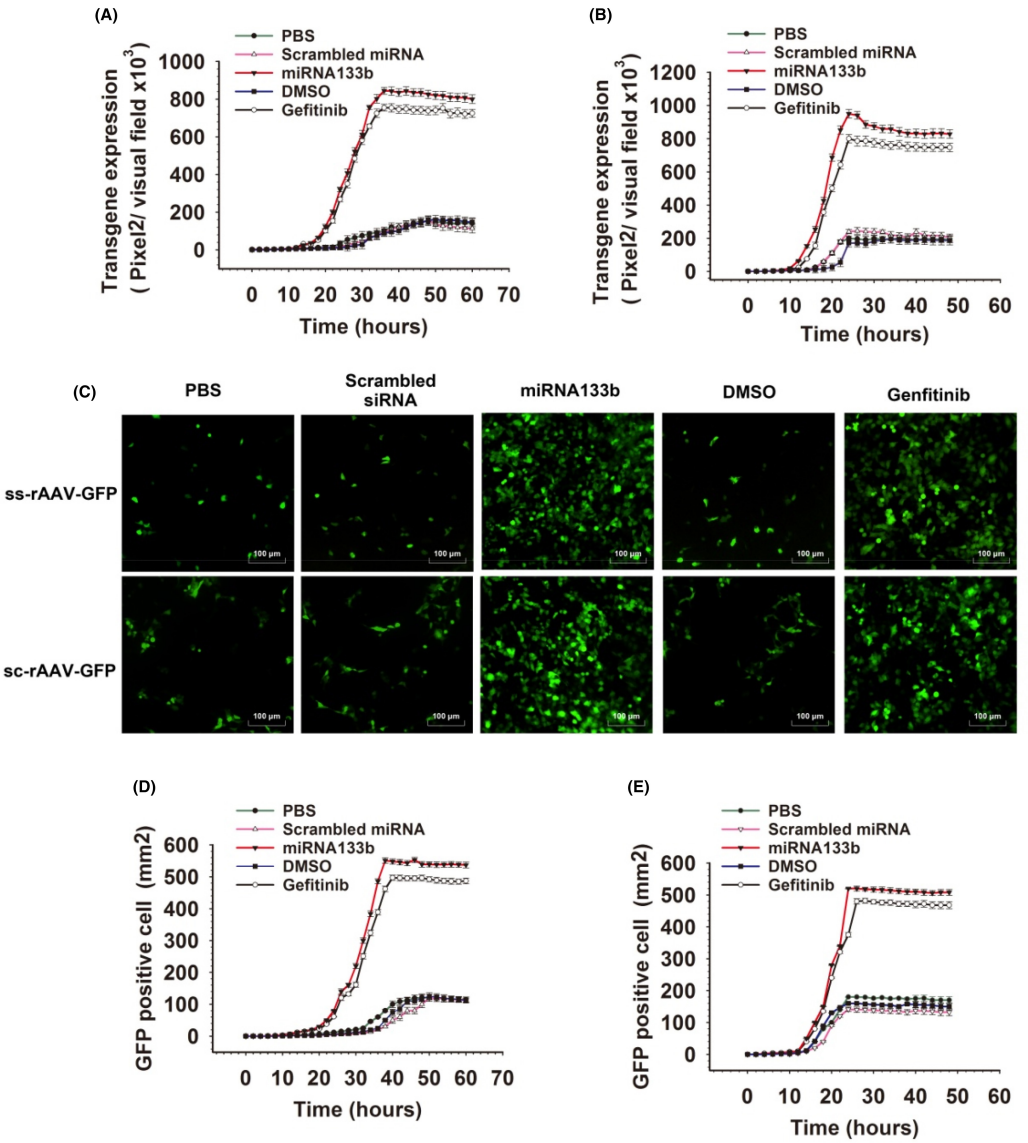
rAAV2‐regulated transgene expression in HeLa cells with/without miRNA133b and gefitinib pretreatment after single‐stranded ss‐rAAV2‐GFP or sc‐rAAV2‐GFP vectors transfection. The GFP fluorescence intensity of ss‐rAAV2‐GFP (A) or sc‐rAAV2‐GFP (B) transduced cells was assayed every 2 h after transduction. The GFP fluorescence images of cells transduced with ss‐rAAV2‐GFP and sc‐rAAV2‐GFP (C) at 48 h. The numbers of ss‐rAAV2‐GFP (D) or sc‐rAAV2‐GFP (E) transduced cell per mm^2^ were assayed every 2 h after transduction. All values are shown as means ± SD (*N* = 3).

To test whether miRNA133b is favourable to both ss‐rAAV2 and sc‐rAAV2 transduction, miRNA133b (15 nM) was added to treat HeLa cells, followed by transfection using ss‐rAAV2‐GFP (1500 vg/cell) or sc‐rAAV2‐GFP (1500 vg/cell) vectors. GFP expression level was continuously determined for 58 or 48 h after rAAV2 transduction (Figure 3A,B). miRNA133b treatment increased the GFP expression transduced with ss‐rAAV2‐GFP and sc‐rAAV2‐GFP (Figure 3C), in relative to PBS treatment. The increase was closely related to the increase of GFP‐positive cells (Figure 3D,E). However, scrambled miRNA and DMSO treatment made no impacts on the transduction of rAAV2. To determine whether enhanced rAAV2 transfection was associated with EGFR pathway, gefitinib, an EGFR‐TKI which was approved as first‐line drug for EGFR‐targeted therapy, was tested.^34^, ^35^ Based on Figure 3A,B, inhibition of EGFR signalling significantly upregulated GFP levels in ss‐rAAV2‐GFP and sc‐rAAV2‐GFP vectors, in relative to DMSO treatment. The increased transduction of gefitinib was stable at all of the time points observed, and to a similar extent with miRNA133b. Collectively, EGFR signalling inhibition promoted transfection via ssrAAV2 and sc‐rAAV2 vectors.

Then, this study evaluated the role of these results in reflecting the difference of cellular fate between ssrAAV2 and sc‐rAAV2 vectors. Table 1 shows the effect of miRNA133b on the maximum of GFP expression (Cmax) and the time to Cmax (Tmax) of ss‐rAAV2 and sc‐rAAV2 vectors. Following miRNA133b pretreatment, the Cmax of ss‐rAAV2‐GFP was increased significantly and the Tmax was shortened by approximately 16 h, compared with the scrambled miRNA treatment. In addition, miRNA133b pretreatment significantly increased Cmax, while Tmax of sc‐rAAV2‐GFP was almost unchanged, because sc‐rAAV2 did not require second‐strand synthesis. These data suggest that miRNA133b enhances ss‐rAAV2 transduction via second‐strand synthesis.

**TABLE 1:** Cmax and Tmax of GFP expression for ssrAAV2 and sc‐rAAV2 vectors (*N* = 3). Results are represented by mean ± SD. Diverse letters stand for *p* ≤ 0.05 (significant difference) upon one‐way anova.

Vector	Pretreatment	Cmax (Pixel^2^/visual field × 10^3^)	Tmax (h)
ss‐rAAV2‐GFP	PBS	158 ± 1.9^a^	48.7 ± 1.2^a^
	Scrambled miRNA	152 ± 2.5^a^	49.3 ± 1.2^a^
	miRNA133b	845 ± 5.6^b^	33.3 ± 1.2^b^
	DMSO	153 ± 3.1^a^	48.7 ± 1.2^a^
	Gefitinib	753 ± 0.9^c^	33.3 ± 1.2^b^
sc‐rAAV2‐GFP	PBS	199 ± 1.97^a^	24.7 ± 1.2^a^
	Scrambled miRNA	190 ± 6.17^a^	24.0 ± 2^a^
	miRNA133b	950 ± 5.57^b^	23.3 ± 1.2^a^
	DMSO	202 ± 5.27^a^	23.3 ± 1.5^a^
	Gefitinib	803 ± 6.27^c^	24.0 ± 0^a^

### 3.3 miRNA133b promotes second‐strand DNA synthesis

**FIGURE 4:**
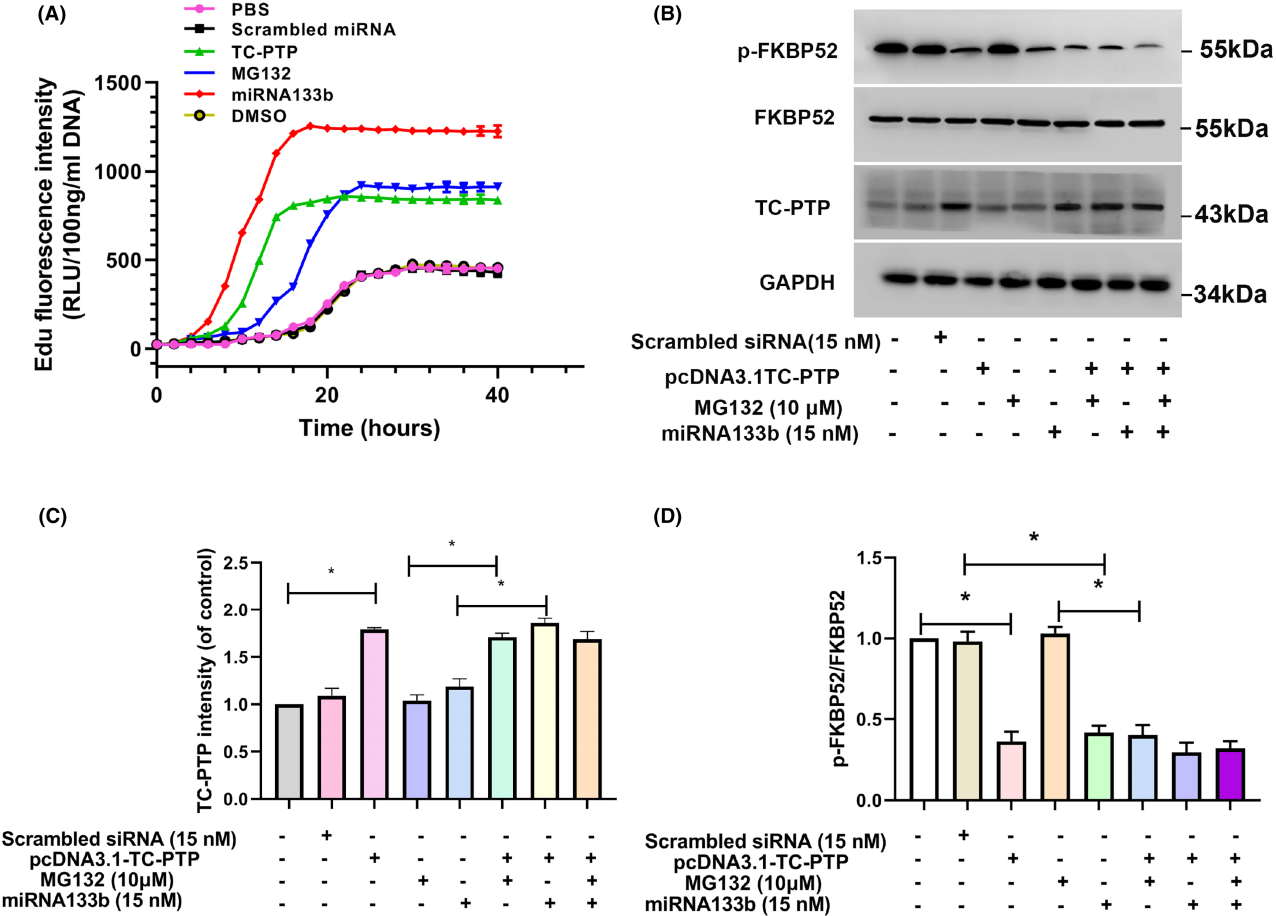
The effect of miRNA133b on the second‐strand synthesis of the ss‐rAAV2. (A) Edu fluorescence spectroscopy analysis of ss‐rAAV second‐strand DNA synthesis in miRNA133b, MG132‐treated or TC‐PTP expression plasmid‐transfected HeLa cells. (B) WB assay on TC‐PTP, FKBP52 and p‐FKBP52 protein expression following miRNA133b, MG132 or TC‐PTP treatment. (C) TC‐PTP protein expression was determined and plotted. (D) p‐FKBP52/FKBP52 ratio was calculated and plotted. Results are shown by mean ± SD (*N* = 3). *p < 0.05.

After entering the nucleus, the single‐stranded genome packaged in ss‐rAAV2 is still transcriptionally inert and should be transformed into double‐stranded DNA (dsDNA) prior to transcription, and this has been considered as the rate‐limiting process for rAAV transduction. This conversion can be achieved by coexisting plus and minus strand annealing in nucleus or through second‐strand synthesis. Based on the above‐mentioned results, we postulated that miRNA33b was related to viral second‐strand DNA synthesis. To characterize the second‐strand rAAV2 DNA synthesis process directly, a time course experiment was performed. To determine second‐strand DNA synthesis, we transfected HeLa cells using ss‐rAAV2 and EdU was used to label viral DNA with active replication. The Hirt procedure after modification was used to modify viral DNA,^36^ followed by click chemistry to tag labelled DNA, and assayed by fluorescence spectroscopy. The results demonstrated that miRNA133b promoted ss‐rAAV2 second‐strand synthesis (Figure 4), similar to TC‐PTP that was reported to improve rAAV2 expression by enhancing second‐strand synthesis.^17^ Both miRNA133b and TC‐PTP increased the second‐strand synthesis, which was monitored by newly synthesized rAAV DNA labelled with Edu, and the Tmax was shortened to reach Cmax of transgene expression (Table 2). Similar increases in the amount of newly synthesized rAAV DNA were observed with miRNA133b, TC‐PTP and MG132 treatment. However, the rate of rAAV DNA synthesis exposed to MG132, an inhibitor recognized to enhance AAV intracellular trafficking, was slower than miRNA133b and TC‐PTP treatment. Previous studies indicated that TC‐PTP dephosphorylates FKBP52 at tyrosine residues, thus negatively regulating EGFR‐PTK signalling and causing efficient viral second‐strand DNA synthesis. WB results show that TC‐PTP and miRNA133b inhibit FKBP52 phosphorylation (Figure 4B–D). Collectively, these results indicate that the promotion of second‐strand synthesis by miRNA133b contributes to the enhanced ss‐rAAV2 transduction, which is a way that is distinct from MG132.

**TABLE 2:** Cmax and Tmax of second‐strand synthesis for ss‐rAAV2 vectors (*n* = 3). Results are represented by mean ± SD. Diverse letters stand for *p* ≤ 0.05 (significant difference) upon one‐way anova.

Vector	Pretreatment	Cmax (RLU)	Tmax (h)
ss‐rAAV2‐GFP	PBS	457 ± 7.6^a^	29 ± 1.2^a^
	Scrambled miRNA	448 ± 3.1^a^	30 ± 1.2^a^
	DMSO	463 ± 5.1^a^	30 ± 1.2^a^
	miRNA133b	1231 ± 13.5^b^	17 ± 1.2^b^
	MG132	922 ± 5.1^c^	29 ± 1.2^a^
	TC‐PTP	880 ± 6.1^d^	17 ± 1.2^b^

### 3.4 miRNA133b improves rAAV2 intracellular trafficking

**FIGURE 5:**
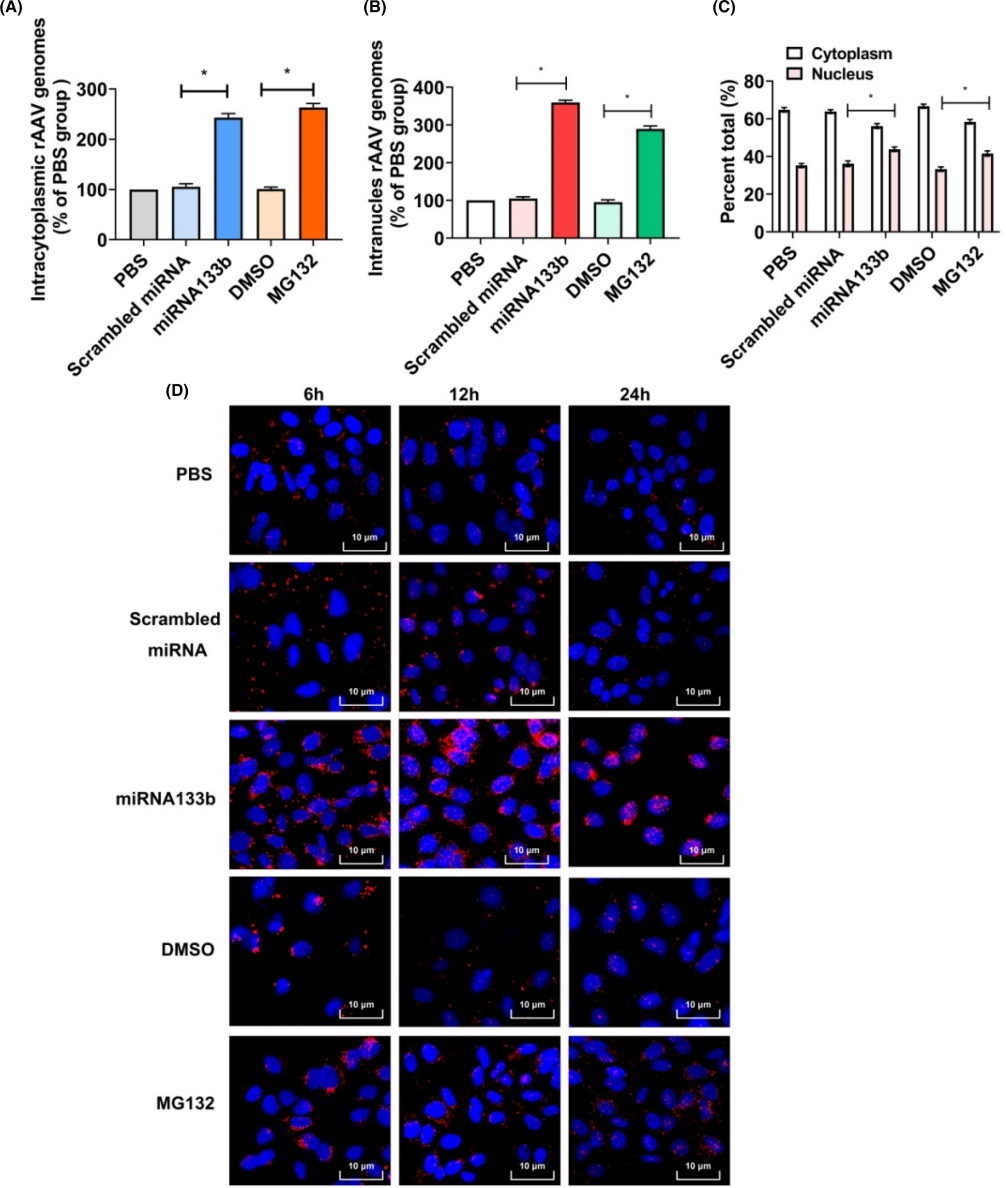
Intracellular trafficking of ss‐rAAV2 vectors following the pretreatment with miRNA133b or MG132. (A) Analysis on cytoplasmic rAAV2 genomes in HeLa cells after Scrambled miRNA, miRNA133b pretreatment or MG132 treatment, (B) analysis on nuclear rAAV2 genomes in HeLa cells after Scrambled miRNA, miRNA133b pretreatment or treatment with MG132, (C) statistical analysis on cytoplasmic and nuclear rAAV2 percentages. Results are shown by mean ± SD (*N* = 3), *p < 0.05. (D) Subcellular localisation of ss‐rAAV2‐TAMRA vectors after the pretreatment with scrambled miRNA, miRNA133b or treatment with MG132. HeLa Cells were fixed at 6, 12 or 24‐h after transduction. The location of ss‐rAAV2‐TAMRA vectors was determined by confocal microscopy. TAMRA and DAPI signals are displayed in red and blue, respectively.

Based on the obtained findings, miRNA133b influenced the second‐strand synthesis of ss‐rAAV2 and its intracellular trafficking. Intracellular trafficking of rAAV has also been indicated as a vital step that influences the efficiency of transduction, and EGFR signalling is involved in rAAV trafficking.^37^ To explore the mechanism of miRNA133b in rAAV2 transfection, we further analysed whether miRNA133b influenced ss‐rAAV2 intracellular trafficking. HeLa cells were pretreated with miRNA133b overnight, and then transduced with ss‐rAAV2‐GFP. MG132 was used as the positive control. At 12 h after transfection, cytoplasmic and nuclear fractions were collected, followed by the quantification of rAAV2 genome DNA using real‐time PCR. Consistent with the previously published studies, ~ 64.7% of input rAAV2 genome DNA existed in cytoplasm of PBS pretreated group, and MG132 pretreatment significantly enhanced its intracellular trafficking efficacy (Figure 5A,B). Pretreatment with miRNA133b improved ss‐rAAV2‐GFP trafficking to the cytoplasm and nucleus up to 2.8 and 3.9 times, respectively, in relative to scrambled miRNA treated cells (Figure 5A,B). Nuclear entry has been identified as the prerequisite of rAAV transfection. In this study, we analysed nuclear entry efficiency after miRNA133b and MG132 treatment. As displayed in Figure 5C, rAAV2 was mostly located in nucleus of miRNA133b or MG132‐mediated cells, compared with the scrambled miRNA or DMSO‐exposed cells. Therefore, nuclear entry and cytoplasmic trafficking of rAAV2 virions are facilitated under miRNA133b treatment.

To confirm the facilitated intracellular trafficking of rAAV2 vectors at virion level, we infected HeLa cells using fluorescently‐labelled rAAV2 to track the corresponding intracellular trafficking, following miRNA133b pretreatment. At 6 h post‐transfection, obvious perinuclear rAAV2 virion accumulation was observed after being exposed to miRNA133b, which increased at 12 and 24 h post transduction; meanwhile, there were fewer remaining in the PBS or scrambled miRNA groups (Figure 5D). In addition, MG132 pretreatment also increased the perinuclear accumulation of rAAV2 virions, which is considered to be associated with decreased capsid degradation in the cytosol.^38^ Therefore, miRNA133b resisted intracellular rAAV2 vector loss in the process of transfection.

### 3.5 miRNA133b decreases ubiquitination of rAAV2 capsid proteins

**FIGURE 6:**
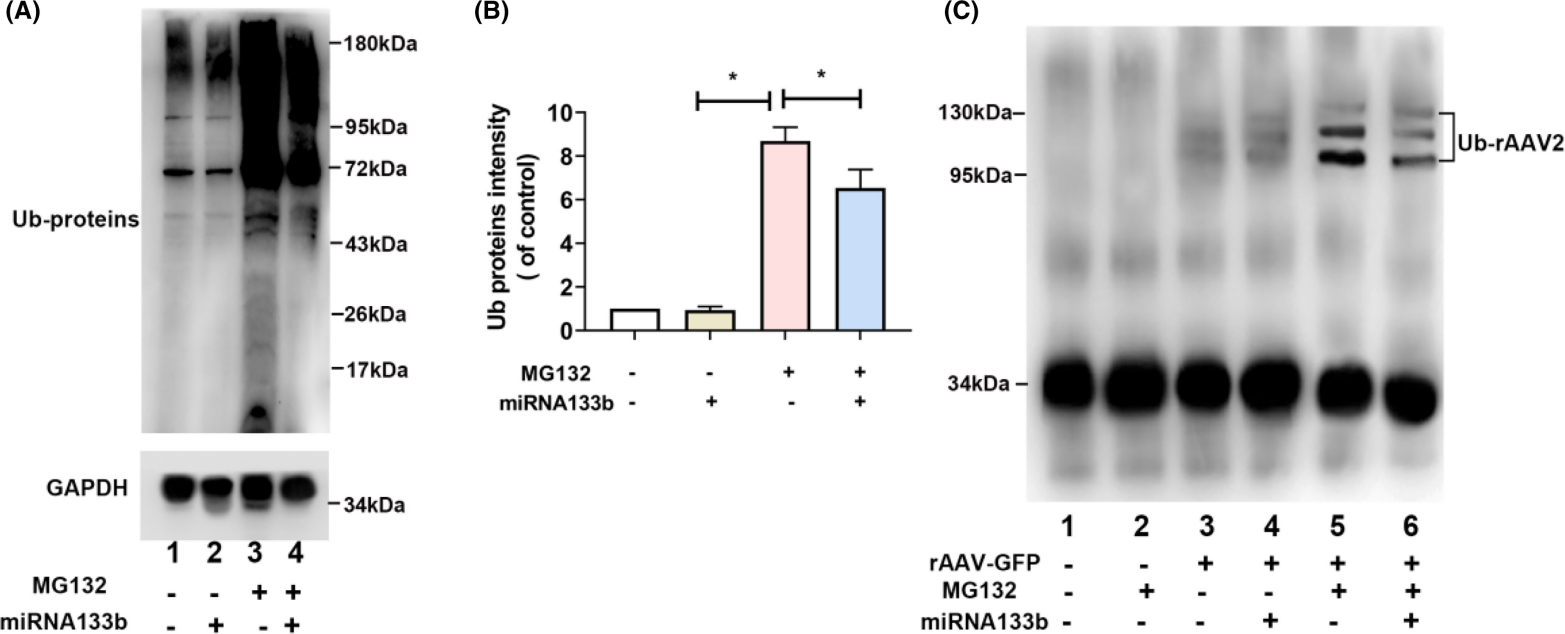
Western blotting (WB) assay on ubiquitinated proteins and ubiquitinated rAAV2 capsid proteins in HeLa cells after miRNA133b transfection with/without MG132 following transduction with ss‐rAAV2 vectors. (A) We prepared whole‐cell lysates in the untreated cells (Lane1) after miRNA133b transfection (Lane 2), MG132 treatment (Lane 3) and cells transfected with miRNA133b transfection following MG132 treatment and incubation using anti‐ubiquitin monoclonal antibody (Lane 4). (B) Ubiquitinated proteins expression was determined and plotted. *N* = 3, *p < 0.05. (C) We prepared whole‐cell lysates in HeLa cells, with/without MG132 treatment after mock‐infection (Lanes 1 and 2), as well as in HeLa cells without (lane 3) and with miRNA133b treatment (Lane 4), with MG132 treatment (Lane 5) or with miRNA133b and MG132 treatment (Lane 6) following transduction with ss‐rAAV2 vectors. Then, anti‐AAV2 capsid antibody A20 was added for immunoprecipitation before WB assay using anti‐ubiquitin (anti‐Ub) monoclonal antibody. IgG, immunoglobulin G.

It is reported that most rAAV2 vectors in the cytoplasm can be decomposed via host cell proteasomal machinery, after the vector escapes from late endosome and the capsid becomes phosphorylated and ubiquitinated.^39^ Inhibitors, such as MG132, can improve intracellular trafficking of rAAV2 due to bypassing proteasome‐mediated degradation pathway. We speculated miRNA133b might play a role in rAAV2 capsid ubiquitination. Two experiments were carried out in this study. First, MG132, or miRNA133b, or both were added to treat cells. Then, we prepared whole‐cell lysates for WB assay using the anti‐Ub monoclonal antibody (Figure 6A). Total ubiquitinated proteins levels were similar between untreated and miRNA133b groups. As our expectation, ubiquitinated proteins were significantly accumulated in MG132 group, which could be explained by inhibiting proteasome activity via MG132. However, the combination of miRNA133b with MG132 significantly reduced MG132‐mediated ubiquitinated protein accumulation. Second, ss‐rAAV2‐GFP was transfected into both treated and untreated cells. At 6 h after transfection, we prepared whole cell lysates, followed by immunoprecipitation using the anti‐AAV2 capsid antibody A20. Meanwhile, anti‐Ub monoclonal antibody was used in WB assay. As presented in Figure 6B, only weak ubiquitinated AAV2 capsid protein signal could be observed in untreated (Lane 3) and miRNA133b (Lane 4) groups; obvious ubiquitinated rAAV2 capsid protein accumulation was found in MG132 group (Lane 5). However, miRNA133b exposure inhibited the MG132‐mediated ubiquitinated rAAV2 capsid protein accumulation (Lanes 6). Based on the above experiments, miRNA133b decreased rAAV2 capsid proteins and total cellular protein levels.

### 3.6 Assessment of transgene expression from the miRNA133b‐regulated vectors

**FIGURE 7:**
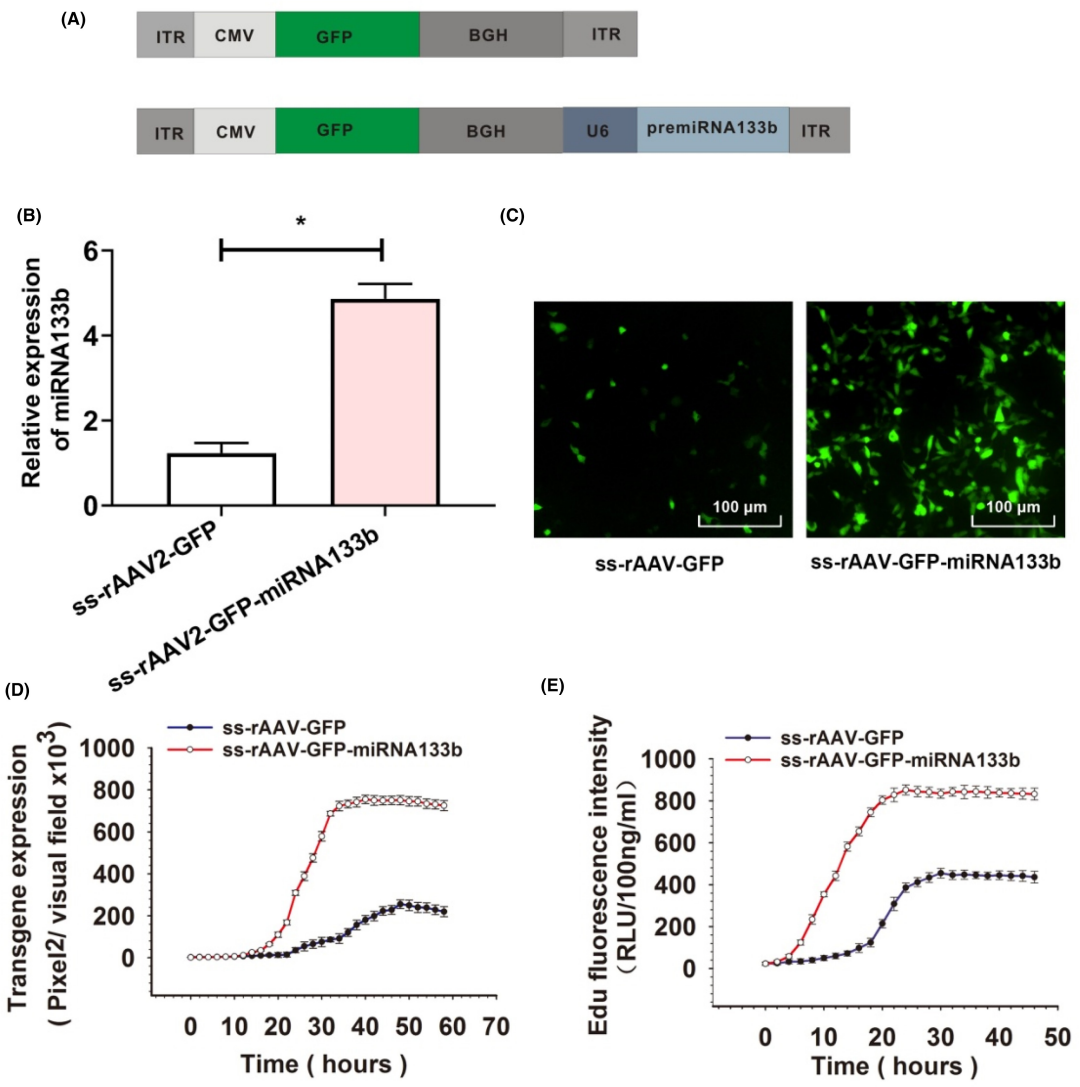
Evaluation of the ss‐rAAV2‐GFP‐miRNA133b vector in vitro. (A) Schematic representation of rAAV2‐GFP and rAAV2‐GFP‐miRNA133b constructs, (B) miRNA133b expression, measured by qPCR, was calculated based on endogenous GAPDH. Results are shown by mean ± SD (*N* = 3). *p < 0.05 versus ss‐rAAV2‐GFP. (C) The GFP fluorescence images of cells transduced with ss‐rAAV2‐GFP or ss‐rAAV2‐GFP‐miRNA133b at 48 h post transduction. (D) The GFP fluorescence intensity of ss‐rAAV2‐GFP or sc‐rAAV2‐miRNA133b transduced cells was assayed every 2 h after transduction. Values indicate the average values in three or more separate assays. (E) Viral second‐strand synthesis at different times after cells transducing with ss‐rAAV2‐GFP‐miRNA133b or ss‐rAAV2‐GFP.

Finally, we constructed a new rAAV2 vector that will express transgene and miRNA133b in cis as well as tested its ability to boost rAAV2 transduction in EGFR‐positive cells. We cloned miRNA133b in previous CMV promoter‐regulated GFP reporter gene‐expressing plasmid backbone. ssrAAV2‐GFP vector that expressed GFP reporter gene alone was used to be the suitable reference. All vectors are displayed in Figure 7A.

HeLa cells were transfected using each of two vectors (MOI = 1000 vg/cell) under the same conditions. Based on Figure 7B, the miRNA133b was effectively expressed by the newly constructed vector. The transgene expression was increased about threefold, and the Tmax of transgene expression was reduced by approximately 20% (Figure 7C,D), when miRNA133b was co‐expressed. Moreover, the extent of second‐strand synthesis was appropriately twofold higher in comparison with ss‐rAAV2‐GFP (Figure 7E). The findings confirmed again that both second‐strand synthesis and cellular trafficking were enhanced due to miRNA133b co‐expression.

**FIGURE 8:**
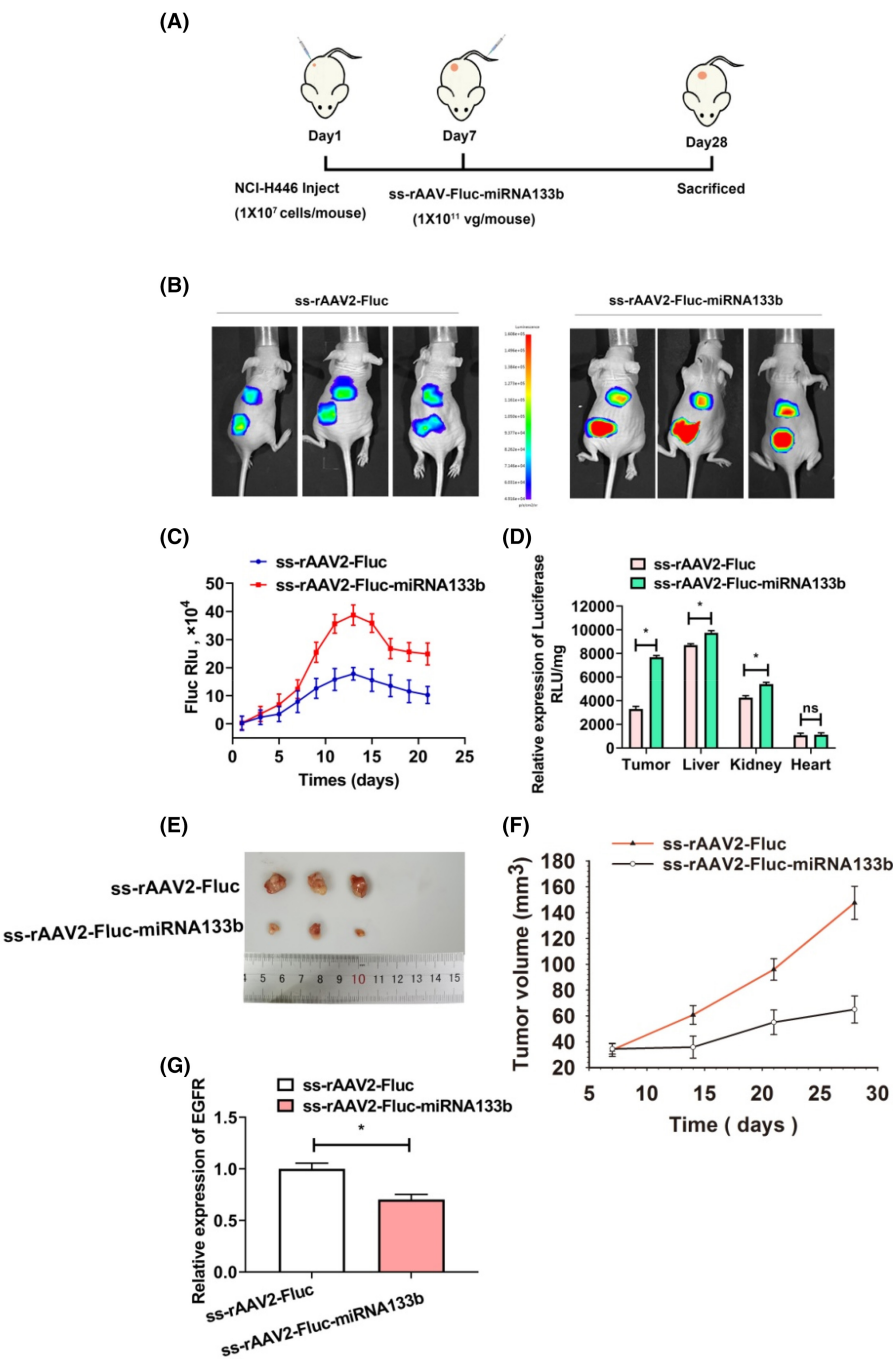
Evaluation of the ss‐rAAV2‐GFP‐miRNA133b vector in vivo. (A) Establishment of orthotopic NCI‐H446 model and treatment schedule. (B) Whole‐body bioluminescence images of BALB/C mice were obtained at 13 days post‐injection. (C) Quantitative data of bioluminescent signals in mouse tumour xenografts at every 2 days after vector administration. (D) Relative luciferase expression in tumour, liver, kidney and heart was analysed. (E) Representative images of tumour tissues in different groups. (F) Volume of tumour in different times. (G) EGFR expression, measured by qPCR, was calculated based on endogenous GAPDH. Results are indicated by mean ± SD (*N* = 3). *p < 0.05, ^ns^ *p* > 0.05.

This study evaluated the in vivo transduction efficiencies of ss‐rAAV‐Fluc‐miRNA133b vector in NSCLC based on the xenograft mouse model. Results indicated a significant increase in Fluc transgene expression with the ss‐rAAV‐Fluc‐miRNA133b vector compared to the ss‐rAAV‐Fluc vector. The ss‐rAAV‐Fluc‐miRNA133b vector did not affect the tissue tropism of rAAV2, but rather enhanced transduction in tumour and liver tissues (Figure 8B–D). In addition, miRNA133b expression was found to significantly inhibit tumour growth (Figure 8E,F) and EGFR expression (Figure 8G), and the luciferase transgene expression significantly elevated after ss‐rAAV‐Fluc‐miRNA133b vector transduction compared with the ss‐rAAV2‐Fluc vector. Interestingly, we found that transgene expression increased in kidney and liver, rather than the heart (Figure 8D). EGFR pathway makes a critical role in liver and kidney development.^40^, ^41^ The EGFR expressed in the liver and kidney influences the transduction of ss‐rAAV2, and the constructed novel ss‐rAAV2‐Fluc‐miRNA133b vector can specifically improve the transduction of rAAV2 in EGFR‐positive tissues. These results demonstrate that ss‐rAAV2‐GFP‐miRNA133b vector specifically increases rAAV2 transduction in EGFR‐positive cells or tissues.

## 4 DISCUSSION

**FIGURE 9:**
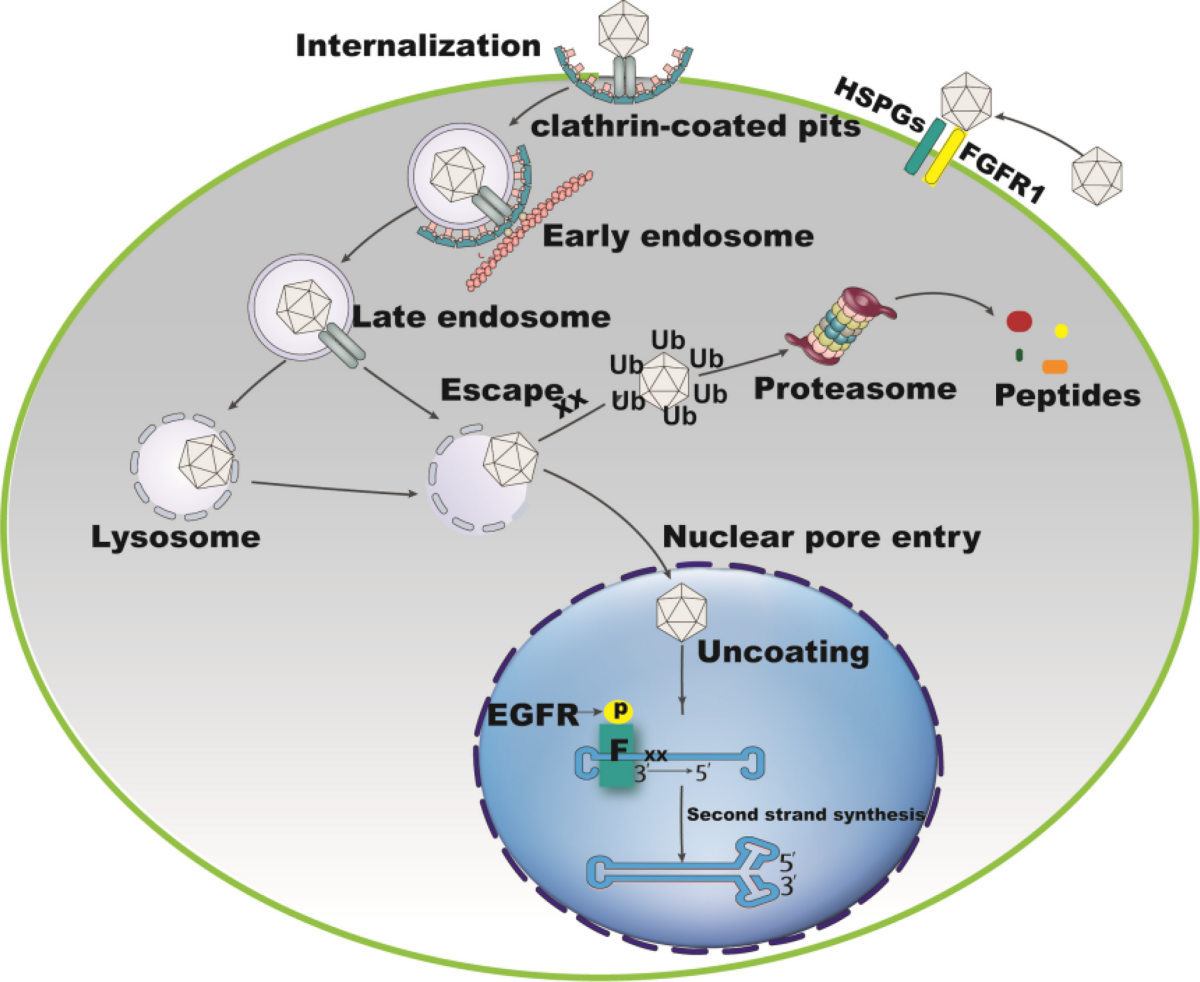
miRNA133b promotes transduction by regulating ss‐rAAV2 ubiquitination and second‐strand DNA synthesis in the EGFR‐positive cell. ^XX^ represents that miRNA133b affects rAAV2 capsids ubiquitination or the viral second‐strand DNA synthesis. P, phosphotyrosine residues; F, FKBP52; FGFR1, fibroblast growth factor receptor 1; HSPG, heparan sulphate proteoglycan.

In this study, we demonstrate that miRNA133b promoted effects on the rAAV2 transduction of EGFR‐positive cells. The main mechanism refers to that miRNA133b inhibits rAAV2 capsid ubiquitination and promotes second‐strand synthesis by regulating the EGFR signalling pathway. Based on the data that miRNA133b promotes rAAV transduction, this study proposed a model to illustrate the interaction between miRNA33b and EGFR‐TPK pathways, aiming to regulate the intracellular transport of rAAV2 vectors and second‐strand synthesis of vector DNA (Figure 9). To successfully transduce a target cell, it is necessary for the rAAV vector to overcome several cellular roadblocks. An initial step of rAAV transduction is to bind to its primary receptor and/or co‐receptors,^42^ followed by endocytosis. During intracellular trafficking, the endocytosed rAAV is transported from the early endosome to late endosome to trans‐Golgi network, prior to nuclear entry. The phospholipase A2‐mediated escape of rAAV into cytosol is vital for nuclear entry. However, this step also provides a chance for proteasome‐mediated rAAV degradation. miRNA133b deceases rAAV2 proteasomal degradation by inhibiting ubiquitination of rAAV2 capsid proteins. Then, more rAAV2 vectors have the opportunity for nuclear entry. After entering the nucleus, the single‐strand rAAV genome is released and converted into a double‐strand form, which is considered as another rate limiting step. Phosphorylation of FKBP52 by EGFR‐PTK can bind to the D sequence of AAV ITR, and block the process of second‐strand DNA synthesis. miRNA133b prevents FKBP52 phosphorylation in the tyrosine residues, and inactivates its capacity for D sequence binding. Therefore, the brake of rAAV second‐strand DNA synthesis is released. Undoubtedly, high transduction efficiency of rAAV will be achieved if all the cellular barriers along its path to transduction are removed.

It is generally accepted that the specificity of rAAV is mainly determined by the interactions between the capsid and target cell surface receptors. Natural AAV‐derived vectors show a broad tropism. Therefore, a high dose needs to be applied to transduce target cells and achieve therapeutic transgene expression level. Various strategies on AAV capsid modification have been developed to overcome the specificity limitation of the natural AAV‐derived vectors.^39^ Nevertheless, the capsid modified rAAV vectors may be transduced in target cells, while inefficiency in the intracellular steps can still limit their transduction because rAAV transduction is a multi‐step process. Therefore, a deeper understanding of the intracellular barriers and corresponding strategies to overcome these barriers are essential for a successful rAAV vector design. A recent elegant study revealed that conventionally described rAAV specificity was inaccurate and misleading, because these data were based on high, stable reporter gene expression and failed to report low level or transient transgene expression.^43^ The study showed that rAAV readily transduced various cells at relatively low vector doses, although at low level or transient, suggesting that second‐strand synthesis is rate‐limiting steps for high level and sustained rAAV transduction. This is supported by data provided by Wang et al. that dsDNA intermediates could be efficiently formed in almost every liver cell after rAAV in vivo delivery, while only a small fraction was eventually stabilized for sustained gene expression.^44^ Based on the conventional vector dose of 1 × 10^11^–1 × 10^12^ vector genomes per mouse, 99.9% of all input viral genomes were lost without contributing to rAAV transduction.^45^, ^46^ The capsids of AAV2 escaped from late endosome become ubiquitinated in the cytoplasm and then degraded by the proteasomal machinery. Mah et al hypothesized that the AAV2 capsid proteins were phosphorylated by EGFR‐PTK and supplied the signal for ubiquitination.^14^ miRNA133b expressed by our rAAV vector prevented the capsid from ubiquitination and proteasome‐mediated degradation, which could significantly enhance rAAV intracellular trafficking efficiency.

Another significant challenge in rAAV clinical application refers to host immune response triggered by rAAV capsid, genome and the transgene products.^47^ Although AAV infection is non‐pathogenic in humans, initial exposure induced humoral and cellular immune responses are still reactive to rAAV due to capsid similarity.^48^, ^49^ Pre‐existing anti‐AAV neutralising antibody (NAb) can effectively block rAAV transduction even at low levels.^50^, ^51^ AAV capsid‐derived epitopes presented by professional antigen presenting cells via major histocompatibility complex (MHC) class I pathway can activate CTL,^52^ and cause targeted destruction of transduced cells, as observed in rAAV2 haemophilia B clinical trial.^53^ Moreover, the immune toxicity tends to be correlated with rAAV dose used. Recent reports of deaths of patients who are treated with very high vector doses suggest that the use of more efficient and lower dose of rAAV vectors needs to be explored. Our results showed that rAAV‐miRNA133b transfection efficiency increased by threefold relative to rAAV2 in EGFR cells. Similarly, we found that rAAV‐miRNA133b transgene expression was enhanced not only in tumour tissues, but also in EGFR‐positive tissues including liver and kidney (Figure 8). These results indicate that rAAV‐miRNA133b can be used in spared dose to lower the risk of immune toxicity.

The rAAV‐miRNA133b vectors show the versatility of the vector platform, which is able to be designed to treat EGFR‐positive cell‐related diseases. The liver is still an optimal target of gene transfer research. The smooth long‐run gene transfer into the liver can manage diverse plasma protein insufficiency problems and metabolic diseases including ornithine transcarbamylase deficiency.^54^ However, high doses of AAV capsids to activate T cells to produce antibodies have been demonstrated in multiple clinical researches, which are associated with therapeutic effect loss.^11^, ^53^, ^55^ Lowering the vector dose can effectively reduce the immune response during gene therapy, especially the T‐cell immune response induced by rAAV capsid protein.^56^ We present this gene delivery vector platform that can reduce the dose while maintaining a high transgene in order to ensure the therapeutic effect.

We have developed a gene delivery platform which can enhance rAAV vector transduction in EGFR‐positive cells or tissues. This novel vector delivers its transgene to cell or tissues while significantly enhancing gene delivery and expression in EGFR‐positive cells or tissues in relative to rAAV2. rAAV‐miRNA133b vector might emerge as a promising platform for delivering various transgene to treat EGFR‐positive cell‐related diseases, such as NSCLC.

## AUTHOR CONTRIBUTIONS

**Xiaoping Huang:** Conceptualization (equal); project administration (equal); writing – original draft (equal). **Xiao Wang:** Methodology (equal); validation (equal). **Ling Li:** Formal analysis (equal); investigation (equal). **Qizhao Wang:** Data curation (equal); investigation (equal). **Wentao Xu:** Methodology (equal); software (equal). **Wenlin Wu:** Data curation (equal); resources (equal). **Xiaolan Xie:** Resources (equal). **Yong Diao:** Funding acquisition (equal); project administration (equal); writing – review and editing (equal).

## FUNDING INFORMATION

The National Natural Science Foundation of China (81502687); Natural Science Foundation of the Fujian Province in China (No. 2021J01968, 2019J05094) funded this research with support from the program for Outstanding Young Scientists Training in Fujian Province University.

## CONFLICT OF INTEREST STATEMENT

The authors declare no conflict of interest.

## CONSENT FOR PUBLICATION

The publication of this manuscript has been approved by all authors.

## Supporting information


Data S1.

## Data Availability

All data generated or analysed during this study are included in this published article.
