# Supplementary material for: MiR133b‐mediated inhibition of EGFR‐PTK pathway promotes rAAV2 transduction by facilitating intracellular trafficking and augmenting second‐strand synthesis
Source: J Cell Mol Med. 2023 Jul 19;27(18):2714–29. doi: 10.1111/jcmm.17858 (PMC10494303; doi:10.1111/jcmm.17858)
Supplement: Supplementary file 1 — Data S1. [file JCMM-27-2714-s001.docx]

**Supporting Information**


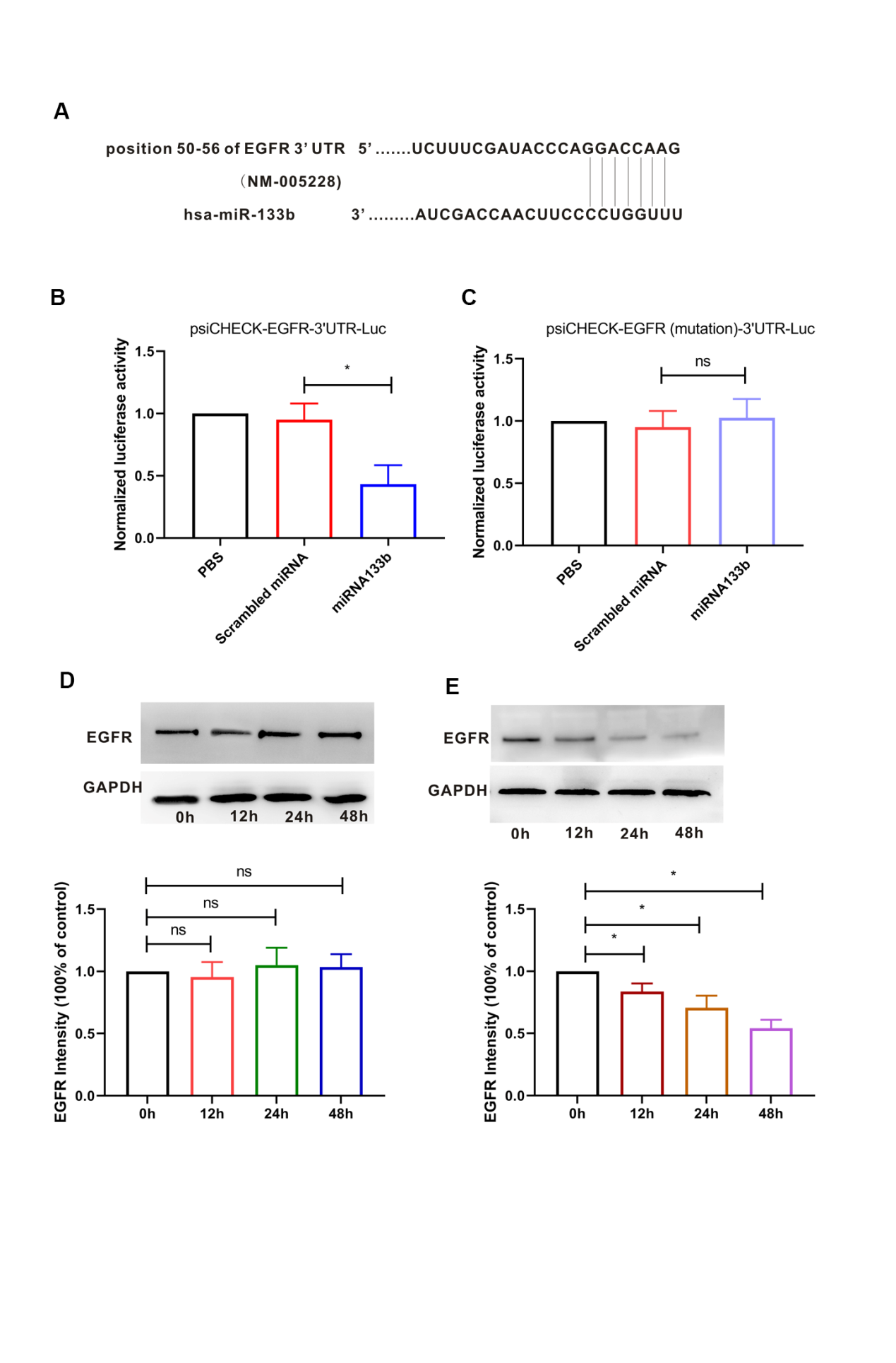


**Fig. S1** MiRNA133b targets EGFR mRNA by binding to the EGFR 3’-UTR. (A) miRNA133b binding sites in EGFR (GenBank ID: NM_005228) 3’UTR predicted by Targetscan 5.2, Luciferase activity was inhibited in the presence of miRNA133b compared with that of Scrambled miRNA. Relative luciferase activity in the Hela cells 48h after transfection of psiCHECK-EGFR-3'UTR-Luc (B) or psiCHECK-EGFR (mutation)-3'UTR-Luc (C) and miRNA133b or scrambled miRNA. Luciferase activity was inhibited in the presence of miRNA133b compared with that of scrambled miRNA, whereas luciferase activity of scramble miRNA was not affected when Hela cell transfection psiCHECK-EGFR-3'UTR-Luc. When HeLa cells were transfected with psiCHECK-EGFR (mutation)-3'UTR-Luc, the luciferase activity of scrambled miRNA and miRNA133b was not affected. Data are shown as mean ± SD of three independent assays, *P < 0.05 vs. scrambled miRNA group. Hela cells were transfected with 15 nM Scrambled miRNA (D) or miRNA133b (E). EGFR expression was detected by western blotting and quantified by densitometry relative to GAPDH expression. Data are shown as the mean ± SD from three independent assays. *P < 0.05 as compared with 0 h.

**Table S1** Sequences of primers used for qPCR analysis of gene expression.

| Gene name | Primer sequences | Accession  number |
| --- | --- | --- |
| miRNA133b | F:5'- gaaccaagtccgtcttcctgaga-3'  R:5'- tagctggttgaaggggaccaaa-3' | MI0000822 |
| Pre miRNA133b | F:5'-cgaaagctt cctcagaagaaagatgccccctg-3' | MI0000822 |
|  | R: 5'- ctgcggccgc tctccaaggactgggcattgc-3' |  |
| EGFR | F:5'- gtgcaaggacacctgccccc-3' | NM_005228 |
|  | R: 5'- cacaggctcggacgcacgag-3' |  |
| U6 gene | F: 5'- ctcgcttcggcagcaca-3' | NC_015438.3 |
|  | R: 5'- aacgcttcacgaatttgcgt-3' |  |
| GAPDH | F: 5'- cgtattggacgcctggtt ac -3' | NC_000012.12 |
|  | R: 5'- ggcaacaacttccactttgc -3' |  |
| GFP | F: 5'- agcagcacgacttcttcaagtcc -3' |  |
|  | R: 5'- tgtagttgtactccagcttgtgcc -3' |  |

**Table S2** Sequences of primers used for *Plasmid* construction.

| TC-PTP | F:5'-tcggatccatgcccaccaccatcgagcgggagttc-3' | M25393.1 |
| --- | --- | --- |
|  | R: 5'-agacgcgtttatagggcattttgctgaaaaaacagtctcc-3' |  |
| Pre miRNA133b | F:5'-cgaaagcttcctcagaagaaagatgccccctg-3' | MI0000822 |
|  | R: 5'- ctgcggccgctctccaaggactgggcattgc-3' |  |
| U6 promoter | F:5'-gaggtaccgagggcctatttcccatgattccttcatatttgcatatacgatacaag-3' |  |
|  | R: 5'-ggaagctttcgtcctttccacaagatatataaagccaagaaatcgaa-3' |  |
